# Proinflammatory Responses of Heme in Alveolar Macrophages: Repercussion in Lung Hemorrhagic Episodes

**DOI:** 10.1155/2013/946878

**Published:** 2013-04-17

**Authors:** Rafael L. Simões, Maria Augusta Arruda, Cláudio Canetti, Carlos H. Serezani, Iolanda M. Fierro, Christina Barja-Fidalgo

**Affiliations:** ^1^Laboratory of Cellular and Molecular Pharmacology, Department of Cell Biology, IBRAG, Avenida 28 de Setembro, 87 Fundos, Vila Isabel, 20551-030 Rio de Janeiro, RJ, Brazil; ^2^Pesquisa e Inovação, Farmanguinhos, Fiocruz, Rio de Janeiro, Brazil; ^3^Instituto de Biofísica Carlos Chagas Filho, Universidade Federal do Rio de Janeiro, 22541-900 Rio de Janeiro, RJ, Brazil; ^4^Department of Microbiology and Immunology, Indiana University School of Medicine, Indianapolis, IN 46202, USA

## Abstract

Clinical and experimental observations have supported the notion that free heme released during hemorrhagic and hemolytic episodes may have a major role in lung inflammation. With alveolar macrophages (AM) being the main line of defense in lung environments, the influence of free heme on AM activity and function was investigated. We observed that heme in a concentration range found during hemolytic episodes (3–30 **μ**M) elicits AM to present a proinflammatory profile, stimulating reactive oxygen species (ROS) and nitric oxide (NO) generation and inducing IL-1**β**, IL-6, and IL-10 secretion. ROS production is NADPH oxidase-dependent, being inhibited by DPI and apocynin, and involves p47 subunit phosphorylation. Furthermore, heme induces NF-**κ**B nuclear translocation, iNOS, and also HO-1 expression. Moreover, AM stimulated with free heme show enhanced phagocytic and bactericidal activities. Taken together, the data support a dual role for heme in the inflammatory response associated with lung hemorrhage, acting as a proinflammatory molecule that can either act as both an adjuvant of the innate immunity and as an amplifier of the inflammatory response, leading tissue injury. The understanding of heme effects on pulmonary inflammatory processes can lead to the development of new strategies to ameliorate tissue damage associated with hemorrhagic episodes.

## 1. Introduction

Numerous cases of acute and chronic pulmonary conditions are accompanied by extravasation of erythrocytes to the lower respiratory tract (lung hemorrhage). These pathological events are frequently associated with marked leukocyte influx and an increase in inflammatory markers [1–7]. In cases of moderate to intense hemolysis that succeed hemorrhagic events, the scavenging of free heme by blood-derived hemopexin or albumin collapses, leading to the accumulation of free heme in the extracellular milieu [3]. It has been previously reported that high expression of haptoglobin, the major protein responsible for the removal of free hemoglobin, reduces tissue injury associated to blood exposure [1]. Accordingly, the induction of heme oxygenase-1 (HO-1) can promote cytoprotective responses in some models of lung injury [8–10]. This stress-inducible enzyme controls the deleterious effect of large amounts of free heme, catabolizing this porfirin in biliverdin, carbon monoxide, and free iron, which are addressed, both directly and indirectly, as cytoprotective agents [10]. These observations support the hypothesis that free heme may be involved in the onset and/or amplification of pulmonary inflammatory responses. 

The proinflammatory role of heme has been extensively demonstrated [11–14]. In mononuclear cells, for instance, heme impairs cytokine release and induces LTB_4_ production in a superoxide dismutase and 5-lypoxigenase-dependent manner, respectively [12, 13]. Furthermore, heme induces peritoneal macrophage necrosis through two distinct and complementary signaling pathways involving autocrine tumor-necrosis-factor- (TNF-) *α* and reactive oxygen species (ROS) production [13]. In addition, heme reduces mice survival and augments cytokine secretion induced by LPS both *in vivo *and* in vitro* [15]. Some of these data suggest that a toll-like receptor (TLR) and a G-protein coupled receptor (GPCR) are molecular targets for free heme resulting in proinflammatory effects [12–14]. However, the impact of heme on alveolar macrophage (AM) physiology has not been investigated.

AMs patrol the alveolar epithelial environment, eliminating microorganisms by phagocytosis/killing, triggering an inflammatory response that can be amplified by the macrophages themselves through the production of inflammatory mediators [16]. Activation of AM by soluble and particulate stimuli can induce an oxidative burst, catalyzed by NOX-2-containing NADPHox enzyme complexes, which generate high amounts of O_2_^−^ through the reduction of molecular oxygen [17]. Cell activation results in phosphorylation and translocation of cytoplasmic components of NADPHox (p40^phox^, p47^phox^, and p67^phox^) to the plasma membrane and consequent association to the membrane-bound subunits (p22^phox^, gp91^phox^) comprising the catalytic active NADPHox [18–21]. Furthermore, the activation of macrophages by bacterial products and cytokines can also induce the activation of inducible nitric oxide synthase (iNOS) with the subsequent production of high amounts of NO, another known microbicidal molecule produced by phagocytes [22–25]. 

Our group characterized free heme as a proinflammatory agent, able to activate polymorphonuclear neutrophils (PMN) and delay the spontaneous apoptosis of these cells by a mechanism that relies on NADPHox-generated ROS [8, 26, 27]. Furthermore, these heme-induced proinflammatory effects involve the activation of the redox-sensitive transcription factor nuclear factor-*κ*B (NF-*κ*B) [8]. NF-*κ*B activation mediates the transcription of cytokines such as IL-1, IL-6, and TNF-*α* [28]. These observations support the idea that free heme could be involved in the development of pulmonary inflammatory responses. However, the role of heme on AM functions requires further investigation. In this study, we demonstrate that heme stimulates ROS and NO production; enhances IL-1*β*, IL-6, and IL-10 release; increases iNOS and HO-1 expression; and potentiates phagocytosis and bacterial killing by rat AM. The mechanisms underlying these effects have also been investigated. The data point to a key role of heme in lung inflammatory processes and may lead to the development of new strategies to ameliorate tissue damage associated with hemorrhagic episodes. 

## 2. Methods

### 2.1. Animals

All experimental procedures used throughout this study were approved by our Institutional Ethics Committee and are in accordance with the National Institute of Health Animal Care Guidelines. Three-month-old Wistar rats free from any infection/pathogen were obtained from animal facilities of the Oswaldo Cruz Foundation (Rio de Janeiro, Brazil) and were kept in a room at a controlled temperature (25 ± 1°C) and with an artificial dark-light cycle (light from 7:00 a.m. to 7:00 p.m.). 

### 2.2. Reagents

Dulbecco modified Eagle medium (DMEM), RPMI 1640, and penicillin/streptomycin/amphotericin B solution were purchased from Life Technologies, Invitrogen (Carlsbad, CA). SDS, antibodies, and type IV horseradish peroxidase (HRP) were purchased from Sigma (St Louis, MO). Heme was purchased from Porphyrin Products (Logan, UT). Compounds requiring reconstitution were dissolved in either ethanol or DMSO. Required dilutions of all compounds were prepared immediately before use, and equivalent quantities of vehicle were added to the appropriate controls.

### 2.3. Cell Isolation and Culture

Rat resident AMs were obtained via *ex vivo* lung lavage as previously described [29] and resuspended in RPMI 1640. Cell suspension density was determined using a hemocytometer, and the appropriate number of AM was allowed to adhere in flat-bottom 6-, 24-, and/or 96-well plates (BD Biosciences) for 1 h at 37°C in a 5% CO_2_ atmosphere. Nonadherent cells were removed by washing the monolayers with serum-free medium, resulting in more than 99% of adherent cells identified as AMs by use of a modified Wright-Giemsa stain (Diff-Quik; American Scientific Products, McGraw Park, IL). Adherent macrophage monolayers were cultured overnight in DMEM with 10% FBS (HyClone). The cells were washed and the medium was replaced by DMEM without serum 20 min before assays. None of the treatments affected AM viability, as determined by MTT (3-(4,5-Dimethylthiazol-2-yl)-2,5-diphenyltetrazolium bromide) reduction assay and Trypan Blue dye exclusion (data not shown).

### 2.4. ROS Production Assays

AMs were suspended in Hank's balanced salt solution (HBSS) and placed in a white 96-well plate (2 × 10^5^ cells/well, final volume 200 *μ*L). Cells were then pretreated or not with DPI (10 *μ*M) or apocynin (10 *μ*M) for 15 min at 37°C in 5% CO_2 _ atmosphere and loaded with luminol (500 *μ*M). Next, cells were stimulated with heme (1–30 *μ*M) or PMA 10 ng/mL, a known NADPHox inducer as a positive control. Cells that remained unstimulated were considered control group. The plate was placed in an Envision plate reader (PerkinElmer) and ROS production was accessed by measuring the chemiluminescence in each well for 20 seconds, performing time kinetics every 10 min for an hour [30].

### 2.5. Immunoprecipitation

AMs (3 × 10^6^ cells/mL) were plated in 6-well tissue culture dishes and incubated with heme (10 *μ*M) for different periods of time at 37°C. After incubation, the macrophage monolayers were lysed in lysis buffer containing 50 mM tris-HCl (pH 7.4), 150 mM NaCl, 1.5 mM MgCl_2_, 1.5 mM EDTA, Triton X-100 (1% v/v), glycerol (10% v/v), aprotinin (10 *μ*g/*μ*L), leupeptin (10 *μ*g/*μ*L), pepstatin (2 *μ*g/*μ*L), and 1 mM PMSF. Lysates (2 *μ*g/*μ*L) were incubated overnight at 4°C with an anti-p47 antibody (1 : 200; Santa Cruz Biotechnology). Then, protein A/G agarose (20 *μ*L/mg protein; Santa Cruz Biotechnology) was added and samples were incubated at 4°C under rotation for 2 h [31]. The content of total and phosphorylated protein was analyzed by immunoblotting.

### 2.6. Determination of TNF-*α*, CINC, IL-1*β*, IL-10, and IL-6 Levels

AMs (1 × 10^6^/mL) were plated in 6-well tissue culture dishes and incubated with heme (3–30 *μ*M) for 3 h at 37°C, followed by immediate freezing in an ice bath to stop the reaction and centrifuged at 10,000 rpm for 5 min at 4°C. The supernatants were collected and TNF-*α*, CINC, IL-1*β*, IL-10 and IL-6 levels were determined by an enzyme-linked immunosorbent assay (ELISA). Briefly, microtitre plates (Nunc-Maxisorb) were coated overnight at 41°C with a specific antibody. After blocking the plates, 50 *μ*L of samples were added in triplicate and maintained at room temperature for 2 h. Standard curves were obtained by serial dilution of rat TNF-*α*, CINC, IL-1b, IL-10, and IL-6. Sheep anti-TNF-*α*, CINC, IL-1b, IL-10, and IL-6 biotinylated polyclonal antibodies were added (1 : 500) followed by incubation at room temperature for 1 h. Finally, 100 *μ*L of avidin-HRP (1 : 5000) was added to each well and, after 30 min, the plates were washed and the color reagent OPD (40 mg/mL; 50 *μ*L/well) was added. After 15 min, the reaction was terminated with H_2_SO_4 _ (1 mM; 50 *μ*L/well) and the optical density measured at 490 nm. The results were obtained by comparing the optical density with standard curves [32]. 

### 2.7. NO Production

NO release by AM was determined by the accumulation of the stable end-product nitrite in cell-free culture supernatants using a modified Griess reaction method [32]. Briefly, AMs (2 × 10^6^ cell/mL) were incubated in RPMI-1640 medium (Sigma, St. Louis, MO) supplemented with 10% heat inactivated FCS (Fetal Calf Serum; Invitrogen, Carlsbad, CA), 100 U/mL penicillin, and 100 mg/mL streptomycin. After 8 h incubation with 5%  CO_2_ and 95%  O_2_ at 37°C, samples were made to react with the Griess reagent (1% sulphanilamide in 5% phosphoric acid, 0.1% naphtylethylenodiamine) at room temperature for 10 min. Thereafter, the nitrite concentration was measured by absorbance at 490 nm, referring to the standard curve of sodium nitrite (dissolved in culture medium solution) within a concentration range.

### 2.8. Preparation of Cell Extracts

AM (1 × 10^6^/mL) were seeded in 6-well tissue culture dishes, incubated with different concentrations of heme (3–30 *μ*M) for different periods of time at 37°C, followed by immediate freezing in an ice bath to stop the reaction, and centrifuged at 10,000 rpm for 5 min at 4°C. To obtain the whole cell extracts, AMs were resuspended in proper lysis buffer (50 mM MES, pH 6.4, 1 mM MgCl_2_, 10 mM EDTA, 1% Triton X-100, 1 *μ*g/mL DNAse, 0.5 *μ*g/mL RNAse, and the following protease inhibitors: 1 mM phenylmethyl-sulfonyl fluoride (PMSF), 1 mM benzamidine, and 1 *μ*M soybean trypsin inhibitor. Proteins present in whole cell extracts were recovered by acidic precipitation and dissolved in 1% (v/v) SDS solution [31]. Immunoblots were developed as described below. The total protein content in the cell extracts was determined by the method of Bradford [33].

### 2.9. Immunoblotting

Cell lysates were denatured in LaemmLi's sample buffer (50 mM tris-HCl, pH 6.8, 1% SDS, 5% 2-mercaptoethanol, 10% glycerol, 0.001% bromophenol blue) and heated in a boiling water bath for 3 minutes. Samples (30 *μ*g total protein from cell extracts) were resolved by SDS-PAGE and proteins were transferred to PVDF membranes. The membrane was blocked with 5% fat-free milk in Tris-buffered saline (TBS) containing 0.1% Tween-20 for 1 h, washed 3 times, and then probed with anti-HO-1 (1 : 1000), anti-p47 (1 : 1000), anti-pSer (1 : 1000), anti-iNOS (1 : 500), anti-arginase-1 (1 : 100-), anti-tubulin (1 : 1000), and anti-actin (1 : 1000) antibodies for 2 hours. After that, the membrane was washed and incubated with a horseradish-peroxidase- (HRP-) conjugated sheep anti-rabbit secondary antibody (1 : 10000; Amersham Pharmacia Biotech, Piscataway, NJ). Bands were visualized using the enhanced chemiluminescence system (ECL, Amersham; Arlington Heights, IL) [31]. Relative band densities were determined by densitometric analysis using National Institutes of Health Image software, and the ratios calculated. In all instances, band density values were corrected by subtraction of the background values.

### 2.10. Immunocytochemistry

AMs (1 × 10^6^ cells) plated onto cytopreps in RPMI 1640 were pretreated with DPI (10 *μ*M) and incubated in the presence of heme (10 *μ*M) for 1 or 2 h at 37°C. LPS (1 *μ*g/mL) was used as positive control. After incubation, cells were fixed with 4% paraformaldehyde and 4% sucrose in PBS for 20 min at room temperature. Cells were then permeabilized for 5 min in PBS containing 0.2% Triton X-100, washed with PBS, and incubated with a rabbit anti-p65 subunit of NF-*κ*B antibody (1 : 50), for 1 h at room temperature. Following this step, cells were incubated with a Cy3-conjugated anti-rabbit antibody. Cytopreps were mounted on a slide using a solution of *N*-propyl gallate (20 mM) and glycerol (80%) in PBS before examination under an epifluorescence microscope (Olympus Model Bx40F4) [31].

### 2.11. Microcolorimetric Erythrocyte Phagocytosis Assay

The phagocytosis of sheep red blood cells (sRBCs—Biocampo, Nova Friburgo, RJ, Brazil) by rat AMs was assessed as previously described [34]. Briefly, AMs were plated at a density of 2 × 10^5^ cells/well in 96-well plates. sRBCs were opsonized with a subagglutinating concentration of rabbit polyclonal anti-sRBC IgG (Cappel Organon Teknika, Durham, NC) [35]. AMs were washed twice with warm DMEM and preincubated with heme (0.1–30 *μ*M). Following treatment, opsonized sRBCs were added at a ratio of 20 : 1 (sRBC : AM) and the cocultures were incubated for further 90 min at 37°C. Cells were washed five times with PBS to remove noningested erythrocytes and treated with 100 *μ*L of 0.3% SDS in PBS for 10 min, and 100 *μ*L of 3′ 3′ 5′ 5′-tetramethyl-benzidine (TMB) was added to each well as a chromogen. Following 5 min incubation (at 22°C) in the dark, the absorbance at 450 nm was evaluated with an automated reader (Envision plate reader, PerkinElmer). A standard curve was derived for each experiment, made through serial dilutions of known amounts of sRBCs, SDS solution, and TMB. The number of sRBCs per well was derived from absorbance data at 450 nm using the standard curve. The absorbance represented the number of sRBCs in an experimental well (ingested plus adhered sRBCs). Experiments were performed in heptaplicates.

### 2.12. Tetrazolium Dye Reduction Assay of Bacterial Killing

 Ingested bacteria viability was quantified using a tetrazolium dye reduction assay, as described elsewhere [36]. Rat AMs (2 × 10^6^/mL), prepared as described above, were seeded in duplicate on 96-well tissue culture dishes. *Klebsiella pneumoniae* strain 43816, serotype 2, was obtained from the American Type Culture Collection (Manassas, VA), and aliquots were grown in tryptic soy broth (Difco, Detroit, MI) for 18 h at 37°C. *K. pneumoniae* were opsonized with 3% anti-*K. pneumoniae* rat-derived immune serum. Cells were then treated with heme (3–300 *μ*M) for 20 min and infected with a 0.1 mL suspension of opsonized *K. pneumoniae* (1 × 10^7^ colony-forming units (CFU)/mL; multiplicity of infection (MOI), 50 : 1) for 30 min to allow phagocytosis to occur. Cell monolayers were washed, tetrazolium dye reduction assay was performed, and bacterial killing was assessed by the intensity of the absorbance at 595 nm, which is directly proportional to the number of viable bacteria associated with the macrophages. Results were expressed as the percentage of survival of ingested bacteria. Preliminary experiments compared and validate this colorimetric assay with a conventional CFU-based (serial dilution) assay, and similar results were obtained (data not shown).

### 2.13. Statistical Analysis

The data are representative of different experiments and expressed as mean ± standard deviation (S.D.). The values of different treatments were compared using Student's *t*-test and ANOVA, followed by *Bonferroni t*-test for unpaired values. 

## 3. Results

### 3.1. Heme Induces ROS Production by AMs in a NADPHox-Dependent Manner

**Figure 1:**
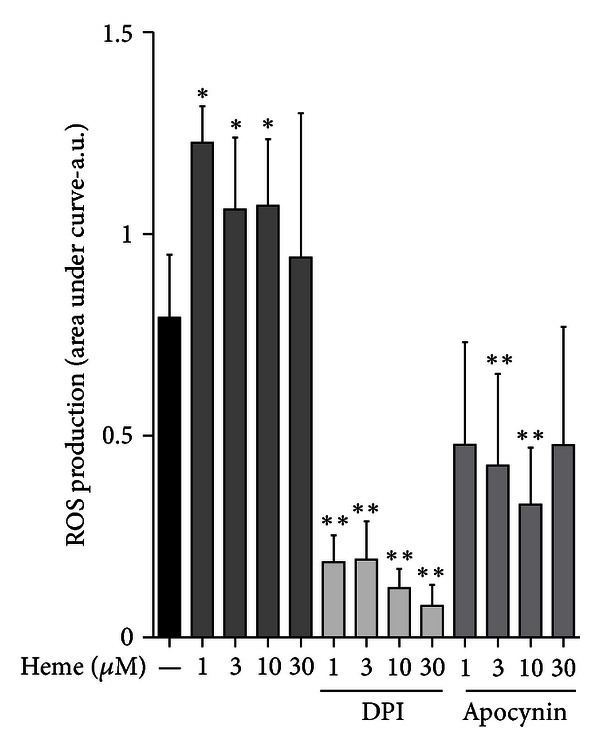
Heme induces ROS production by AM. Rat AMs (2 × 10^5^/mL) were placed in a white flat-bottom 96-well plate. Cells were pretreated with DPI (10 *μ*M) and apocynin (10 *μ*M) and then stimulated or not with heme (1–30 *μ*M) for 1 hour in the presence of luminol as a chemiluminogenic probe (500 mM). Absorbance intensity at 595 nm was directly proportional to ROS production by AM. DPI and apocynin alone had no effect. The data shown are representative of three independent experiments. The mean ± S.D. is presented. *P < 0.05 when compared with controls. **P < 0.05 when compared to cells treated with heme.

**Figure 2:**
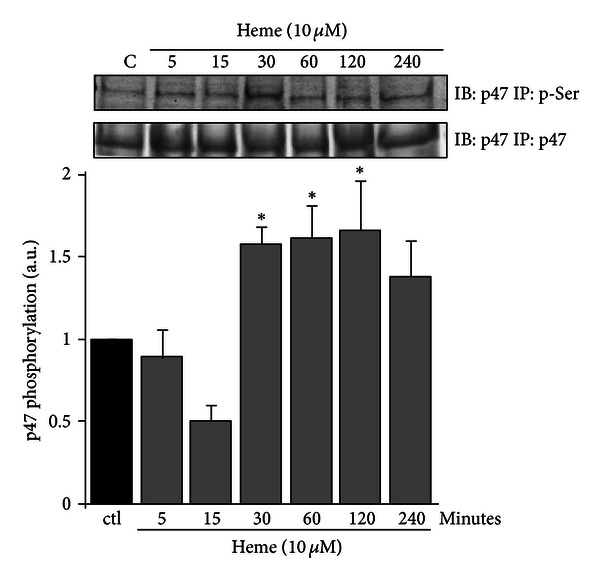
Heme induces p47^phox^ phosphorylation in AM. Rat AMs (3 × 10^6^/mL) were cultured in the absence or presence of heme (10 *μ*M) for different periods of time. Incubations were terminated by the addition of lysis buffer, and lysates were subjected to immunoprecipitation of p47^phox^ and immunoblots with an anti-p47^phox^ and p-serine antibodies were performed. The data shown are representative of three independent experiments. The mean ± S.D. is presented. *P < 0.05 when compared with controls.

The activation of AM caused by exposure to different stimuli might result in the production of ROS that can be measured using luminol as a chemoluminescent probe. We observed that heme triggered robust accumulation of ROS throughout the experiment (1–60 min, Figure 1). Rather than being nonspecific, heme-generated ROS depended on NADPHox activation. Pretreatment of AM with DPI (10 *μ*M), an inhibitor of NADPHox activity, or with apocynin (10 *μ*M), which inhibits the translocation of p47^phox^ to the membrane, both significantly reduced ROS production by AM. Supporting a role for NOX2 in heme-induced ROS production, Figure 2 shows that heme (10 *μ*M), after 30 min of incubation, induces p47^phox^ phosphorylation in AMs.

### 3.2. Heme Modulates Cytokine Secretion in AMs

**Figure 3:**
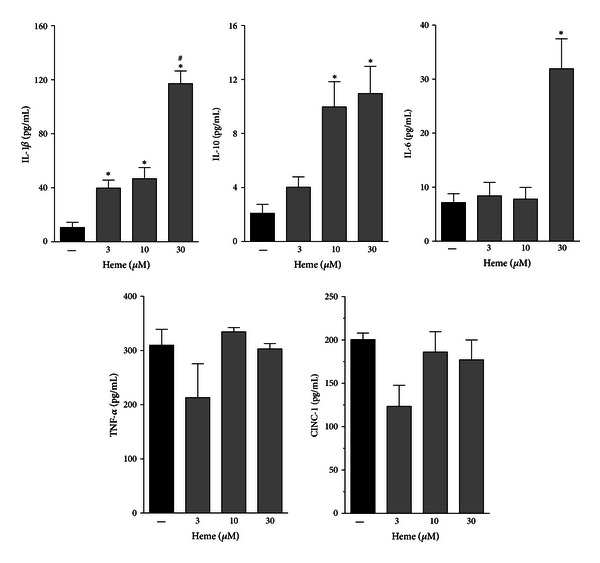
Heme modulates cytokine secretion in AM. Rat AMs (1 × 10^6^/mL) were treated with different concentrations of heme (3–30 *μ*M) for 3 h. The culture supernatants were subsequently collected and analyzed for IL-1*β*, IL-6, IL-10, TNF-*α*, and CINC-1 secretion by ELISA. The data shown are representative of three independent experiments. The mean ± S.D. is presented. *P < 0.05 when compared with untreated cells; ^#^ *P* < 0.05 when compared to heme 3 and 10 *μ*M.

We investigated the profile of cytokine release by AM stimulated with different concentrations of heme (3–30 *μ*M). The treatment with heme (Figure 3) markedly induced IL-1*β* and IL-6 release, peaking at 30 *μ*M. In contrast, heme did not affect TNF-*α* or CINC-1 secretion. Interestingly, heme (10–30 *μ*M) was also able to induce the secretion of IL-10, an anti-inflammatory cytokine. 

### 3.3. Heme Induces NO Production by AM

**Figure 4:**
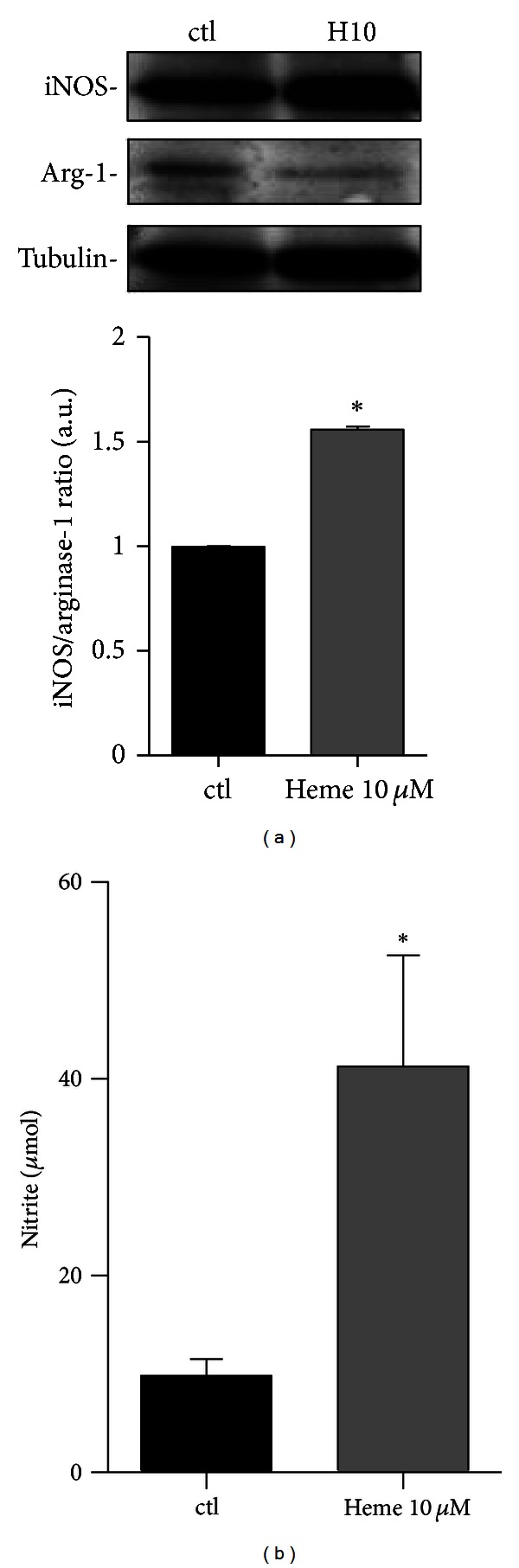
Heme induces NO production by AM. (a) Rat AMs (1 × 10^6^/mL) were pretreated with heme (10 *μ*M) for 8 hours. Incubations were terminated by the addition of lysis buffer and lysates were subjected to immunoblotting with anti-iNOS, anti-arginase-1, and anti-tubulin antibodies. Results are demonstrated as the iNOS/Arg-1 ratio. Tubulin was used as a loading control. (b) Rat AMs (2 × 10^5^/mL) cultured in 96-well plates were pretreated with heme (10 *μ*M) for 24 hours. NO production was assessed by the Griess reaction and read on an Envision plate reader, as previously described [29]. The data shown are representative of three independent experiments. The mean ± S.D. is presented. *P < 0.05 when compared with controls.

The treatment with heme (10 *μ*M) triggers an increase in iNOS expression with the concomitant decrease in arginase expression, an enzyme that catabolizes arginine, the main substrate for NOS (Figure 4(a)). The increase of iNOS/arginase-1 ratio was accompanied by a significant NO production by AM, as shown in Figure 4(b).

### 3.4. Heme-Induced HO-1 Expression in AM is NADPHox Dependent

**Figure 5:**
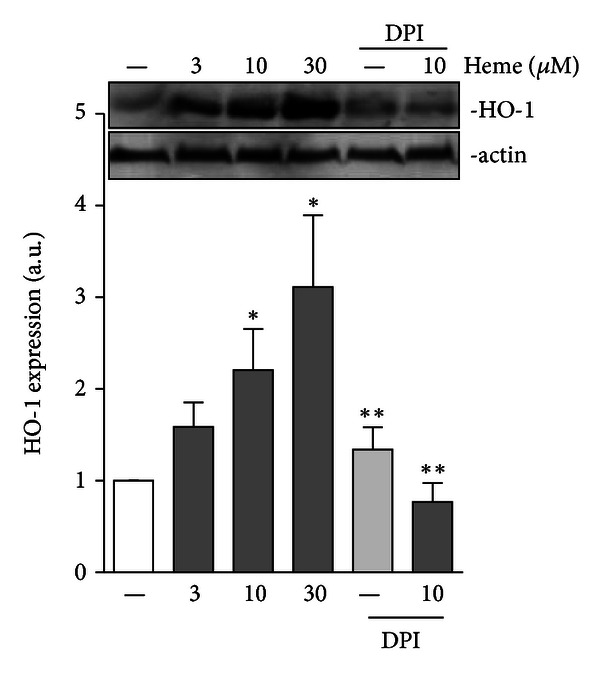
Heme-induced HO-1 expression is NADPH dependent. Rat AMs (1 × 10^6^/mL) were pretreated with DPI (10 *μ*M) followed by incubation in the absence or presence of heme (10 *μ*M) for 24 h. Incubations were terminated by the addition of lysis buffer and lysates were subjected to immunoblotting with anti-HO-1 and anti-actin antibodies. Results are demonstrated as the HO-1/actin ratio. The data shown are representative of three independent experiments. The mean ± S.D. is presented. *P < 0.05 when compared with controls. **P < 0.05 when compared to cells treated with heme.

 The treatment of AM with heme (10–30 *μ*M) induces expression of HO-1, as shown in Figure 5. Furthermore, heme-induced HO-1 expression is both concentration and NADPHox dependent, as this phenomenon was significantly inhibited by pretreatment of the cells with DPI, a NADPHox inhibitor (Figure 5).

### 3.5. Heme Induces the Nuclear Translocation of NF-*κ*B in AMs

**Figure 6:**
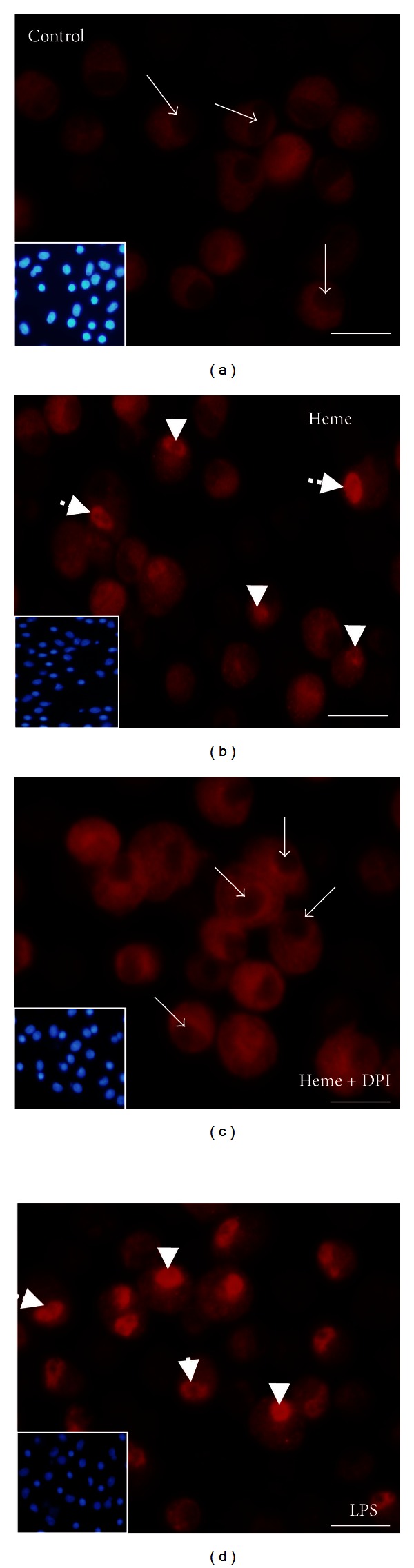
Heme induces NF-*κ*B nuclear translocation in AM. Rat AMs (1 × 10^6^/mL) were treated with heme (10 *μ*M) for 1 hour. NF-*κ*B nuclear content was assessed through immunocytochemistry using a Cy3-conjugated anti-p65 subunit antibody. LPS (1 *μ*g/mL) was used as positive control. The data shown are representative of three independent experiments. *Arrows*: nuclei without NF-*κ*B. *Arrowheads*: nuclei with NF-*κ*B.

To investigate whether heme activates NF-*κ*B in AM, cells were stimulated with heme (10 *μ*M) and the nuclear translocation of the p65 NF-*κ*B subunit was accessed by immunocytochemistry. As shown in Figure 6, heme induced the nuclear accumulation of p65, which was comparable to the increase evoked by LPS (1 *μ*g/mL). Moreover, heme-induced NF-*κ*B activation was abolished in the presence of DPI, a NADPHox inhibitor (Figure 6). 

### 3.6. Heme Improves Phagocytosis and Bacterial Killing by AMs

**Figure 7:**
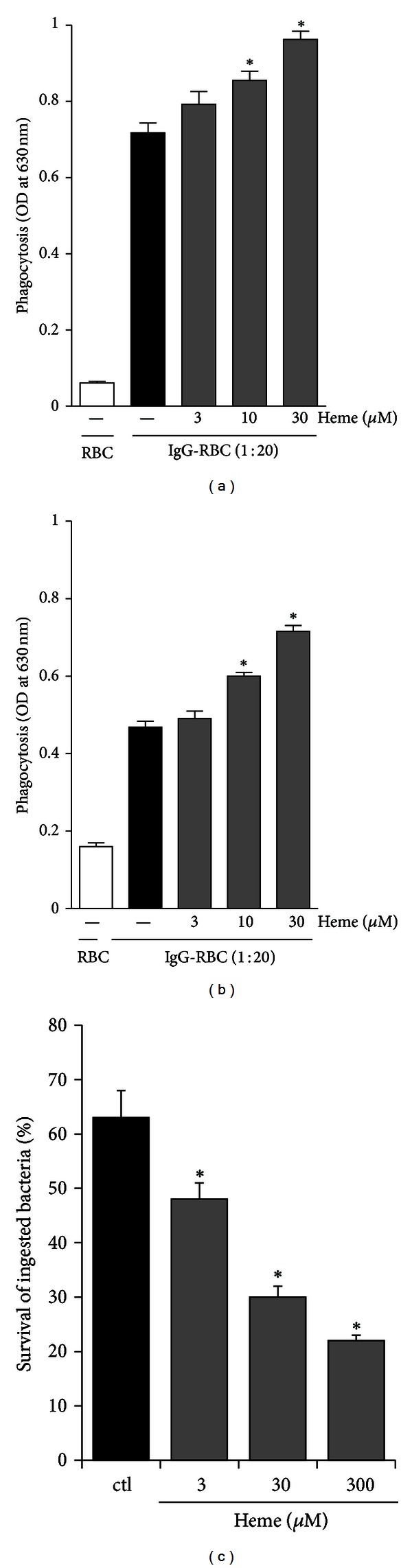
Heme modulates phagocytosis and killing by AM. Rat AMs (2 × 10^6^/mL) were cultured in the absence or presence of heme (3–300 *μ*M) for different periods of time. (a), (b) Following the incubation with heme for 10 min (a) or 24 h (b), IgG-SRBCs were added and cultures were incubated for an additional 90 min at 37°C. Phagocytosis was assessed by a colorimetric assay as described in Section 2. (c) Following the incubation with heme (3–300 *μ*M) for 10 min, cells were infected with opsonized *K. pneumoniae* for 30 min to allow phagocytosis to occur. The intensity of the absorbance at 595 nm is directly proportional to the number of intracellular bacteria associated with the macrophages. Results are expressed as mean ± S.D. of three independent experiments. RBC, macrophage exposed to unopsonized RBC. *P < 0.05 compared with control group.

In order to evaluate the effect of heme on phagocytosis, isolated rat AM were pretreated with heme (0.1–30 *μ*M) for 10 min (Figure 7(a)) or 24 h (Figure 7(b)) before incubation with IgG-opsonized sheep red blood cells (IgG-sRBC). Heme, in a dose-dependent manner, increases FcR-mediated phagocytosis. Both treatments with heme, acute and overnight, induced a consistent increase in IgG-sRBC phagocytosis, which reached maximum levels in a concentration range between 10 and 30 *μ*M. Furthermore, the treatment of AMs with heme (3–300 *μ*M) for 10 min significantly decreased intracellular survival of phagocytosed* Klebsiella pneumoniae* (Figure 7(c)). 

## 4. Discussion

Lung hemorrhagic episodes are accompanied by a severe hemolysis and can occur during pathological states such as chronic bronchitis, cystic fibrosis, lung cancer, pulmonary embolism, pneumonia, tuberculosis, and traumatic injury, resulting in intrapulmonary high levels of free heme [1–3]. Studies have shown heme's proinflammatory properties [8, 9, 12–15, 26, 27], supporting its role as a putative inductor of lung inflammation. AMs are the first line of defense and, in case of lung hemorrhagic episodes, could interact with free heme [3, 4]. We are now showing, for the first time, that heme activates inflammatory and oxidative responses in AM, triggering the production of oxygen and nitrogen species, via NADPHox and iNOS, respectively. Heme also promotes cytokine production and improves phagocytic and microbicidal activities, two phenomena involved in the immune defense against bacterial infection. Coherently with its attributed role as a double-edge sword, displaying both protective and deleterious actions [11, 37], heme also induces HO-1 expression and IL-10 production, two players involved in the control and resolution of inflammation [3, 10, 38, 39]. IL-10 can downregulate the production of TNF-*α*, IFN-*γ*, and certain chemokines [40, 41]. Recently, it was suggested that IL-10 mediates many of its anti-inflammatory effects via upregulation of HO-1 [38]. In our study, we show that heme induced IL-10 production, suggesting that this cytokine could be involved in the increased HO-1 expression in heme-stimulated AM. The overexpression of HO-1 accelerates the removal of free heme at the same time it generates carbon monoxide and biliverdin, agents that may act as anti-inflammatory/cytoprotective molecules [42–45]. Here, we show that HO-1 was detected in AM, only after 24 hours after incubation with heme. This late HO-1 expression may both represent the induction of a counterregulatory, anti-inflammatory response, and a self-preservation mechanism by which these cells prevent continuous cell damage caused by oxidative stress. 

Our group and others have demonstrated that heme signals through PKC activation to activate NADPHox in different cell types [20, 27, 46]. The endogenous production of ROS can modulate redox-sensitive pathways involved in cell functions and fate [47, 48]. In mouse peritoneal macrophages, heme induces TNF-*α* secretion dependently on TLR4 activation and ROS generation, two apparently independent effects [9, 13]. In line with these results, rat AM showed, in a heme-rich milieu, an improvement of their already activated profile, increasing ROS production through activation of NOX2, the NADPHox family member in phagocytes [17], since it was shown that heme triggered p47^phox^ phosphorylation and apocynin, which impairs p47 translocation, inhibited ROS generation. AMs also play a prominent role in orchestrating inflammatory and immune responses through the release of mediators such as cytokines and chemokines [8, 28]. Accordingly, we demonstrate that stimulation of AM with heme induced IL-1*β* and IL-6 release, but did not affect TNF-*α* and CINC-1. These results are in contrast with previous reports in mouse peritoneal macrophages, which demonstrate that heme stimulates TNF-*α* and CXC chemokine production [9]. We believe that these differences are due to the unique physiology of AMs and should be exclusively addressed as AM activation by heme. Supporting this hypothesis, the experimental protocol involves the incubation of adherent AMs overnight, prior to any treatment, which ensures a virtually exclusive AM population. 

We suppose that the disparity in the response to heme displayed by AM and peritoneal macrophages is most probably related to the influence of dramatically different microenvironment surrounding each of these cells. AMs are found in the alveoli and alveolar ducts of the lung, living in an aerobic environment, and are considered ready to act/react to external stimuli, not only as phagocytes but also as potent secretory cells in various lung conditions [39]. These observations are consistent with the results depicted in Figure 3, in which resting AMs (controls) already secrete significant amounts of TNF-*α* and CINC-1, indicating a basal activated profile. Moreover, another indication of the activated state of nonstimulated AMs, is the high expression of iNOS found in these cells (Figure 4). Interestingly, even though the treatment with heme did not significantly increase iNOS expression, it triggered an expressive production of NO in AM. On the other hand, the minor changes in iNOS protein content were accompanied by an important decline in arginase expression, resulting in an increase in the iNOS/arginase ratio. These results indicate that an increased offer of substrate (arginine) can be related to an increased efficiency in the production of NO by iNOS. 

Several observations have shown that once exposed to different microenvironments, macrophages acquire either M1- or M2-polarized phenotypes, which are associated with distinct physiological events, such as inflammation and tissue remodeling, respectively [49]. The M1-phenotype is related to the classical activation by IFN-*γ*, TNF-*α*, or LPS and characterized by a high capacity of antigen presentation and production of proinflammatory cytokines, such as IL-12, IL-1*β*, IL-6, Fc*χ* receptor, and ROS and NO generation, and increased microbicidal activity [25, 49]. In contrast, the M2 phenotype produces high amounts of TGB-*β*, arginase-1, and IL-1ra, but low levels of proinflammatory cytokines [25, 49]. Our results strongly suggest that heme can drive AM to a strong M1 profile that leads to an increase in macrophage microbicidal activity, enhancing both Fc*γ* receptor-dependent phagocytosis and killing of opsonized bacteria. 

Heme was previously shown to activate TLR4 [9] and to synergize with microbial products, such as LPS, increasing cytokine production by mouse peritoneal macrophages, which correlates with NF-*κ*B activation [15]. Furthermore, the same authors demonstrated that generation of ROS by heme was essential for its potentiating effects upon LPS stimulation. These data support the hypothesis that AMs promptly respond to heme. The increased basal levels of TNF-*α* and CINC-1 found in the supernatant of AM would respond to the high expression of iNOS detected in control cells. However, as arginase levels were high in nonstimulated cells, the production of NO would be rather downregulated in AM. On the other hand, the stimulation of AM with heme induces NOX2 activation and the generated ROS would act on activating redox-sensitive pathways, such as NF-*κ*B, which could lead to the decrease in arginase levels, giving rise to a massive NO production, and an improvement in the phagocytic activity. The increased production of ROS and NO, which can react to produce the highly cytotoxic mediator peroxynitrite [50], would be the main factor responsible for the potent microbicidal activity displayed by free heme-stimulated AM. 

**Figure 8:**
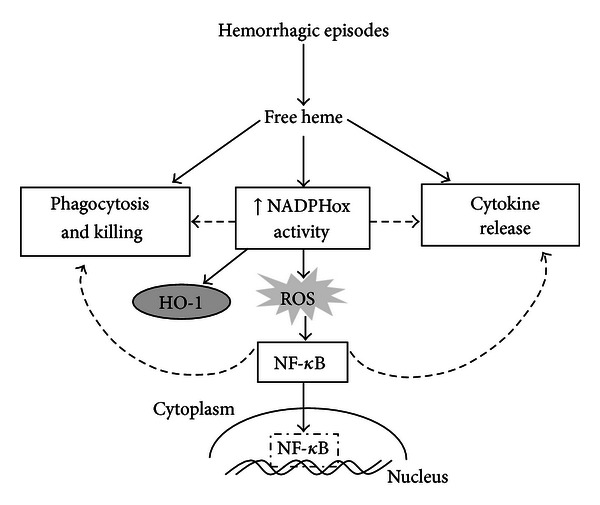
Heme induces a proinflammatory profile in AM. Heme induces NADPHox-dependent ROS production and NF-*κ*B nuclear translocation. These processes can be directly related to NO production, phagocytosis, killing, and cytokine release, functions that are involved in the inflammatory response induced by heme.

Taken together, our data indicate that, in the presence of free heme, AMs, which are possibly primed by their own microenvironment, become activated cells displaying an M1-like profile. It leads to the generation of NADPHox-derived ROS and NO, activation of the redox-sensitive NF-*κ*B pathway, and an increase in cytokine expression, which improves AM antimicrobicidal activities (Figure 8). Recent advances in the study of heme's role as a proinflammatory molecule brings up hope for the development of new strategies to ameliorate acute and chronic inflammation related to hemolytic episodes. However, further investigations are required in order to elucidate how heme interacts with the cell membrane and modulates the activity of other cells of the immune system.
